# Self‐discontinuity, smoker and ex‐smoker identity, and smoking relapse: A multilevel longitudinal study

**DOI:** 10.1111/aphw.70231

**Published:** 2026-09-25

**Authors:** Jérôme Blondé, Juan Manuel Falomir‐Pichastor

**Affiliations:** ^1^ Faculté de Psychologie et des Sciences de l'Education University of Geneva Geneva Switzerland

**Keywords:** ex‐smoker identity, ex‐smokers, self‐discontinuity, smoker identity, smoking cessation, smoking relapse

## Abstract

The present study examined longitudinal associations between smoking‐related self‐discontinuity (i.e. the perception that quitting smoking has disrupted the stability of one's self‐concept over time), smoker and ex‐smoker identity, and smoking relapse. We tested a mediation model in which increases in self‐discontinuity were expected to increase relapse risk through concurrent and prospective increases in smoker identity and decreases in ex‐smoker identity. The model was evaluated in a four‐wave longitudinal study conducted over 1 year among former smokers who had quit within the previous 5 years. Multilevel analyses isolated within‐person associations between self‐discontinuity, identity, and relapse over time while controlling for time‐invariant between‐person differences related to smoking history. Results showed that within‐person increases in self‐discontinuity were indirectly associated with a higher likelihood of relapse at the subsequent wave through their concurrent association with smoker identity, but not ex‐smoker identity. Moreover, no indirect effects emerged when changes in both smoker and ex‐smoker identities were modeled prospectively. Sensitivity analyses provided no evidence for reverse indirect pathways from identity to relapse via self‐discontinuity. However, within‐person increases in both smoker and ex‐smoker identities predicted subsequent increases in self‐discontinuity, suggesting that identity fluctuations may precede feelings of self‐discontinuity rather than the reverse.

## INTRODUCTION

Although many smokers attempt to quit, research has consistently shown that a substantial proportion of quit attempts end in relapse within the first weeks or months following cessation (García‐Rodríguez et al., 2013; Hughes et al., 2014; Zhou et al., 2009). Relapse may therefore be considered a common feature of the quitting process, with estimates suggesting an average of 30 relapse episodes before successful long‐term abstinence (Chaiton et al., 2016). However, reducing relapse remains critically important, as repeated failures may undermine motivation and confidence in one's ability to quit (Partos et al., 2013; Shiffman et al., 2000).

In light of these challenges, research has sought to identify key predictors of relapse risk. Several factors have been documented, including nicotine dependence, smoking cravings, negative affect (e.g. stress and anxiety), and low self‐efficacy (Elfeddali et al., 2012; Herd et al., 2009; Zhou et al., 2009). The present research extended this literature by focusing on identity‐related processes. Specifically, we adopted the premise that smoking cessation involves more than stopping a habitual behavior; it also entails navigating a transition from an identity as a smoker toward a new identity grounded in abstinence.

### Smoking cessation as an identity transition

Recent research has increasingly highlighted the role of identity in smoking behavior (for reviews, see Poole et al., 2022; Tombor et al., 2015). Frequent and sustained tobacco use promotes the development of a smoker identity, whereby smoking becomes a central aspect of the self‐concept (Hertel & Mermelstein, 2016; Shadel & Mermelstein, 1996). Relatedly, tobacco dependence has been shown to be robustly associated with the strength of smoker identity (e.g. Blondé & Falomir‐Pichastor, 2020, 2021; Falomir‐Pichastor et al., 2020).

Smoking cessation also has important identity implications. Once established, a smoker identity strongly influences both the tendency to continue smoking and the difficulty of quitting. Because individuals tend to behave in ways that are consistent with their most salient identities (West, 2006), a strong smoker identity predicts lower motivation to quit, fewer quit attempts, and greater difficulty maintaining abstinence (Falomir & Invernizzi, 1999; Meijer et al., 2015, 2018; van den Putte et al., 2009).

Importantly, quitting involves an identity transition in which individuals progressively disengage from a smoker identity while developing an ex‐smoker (or non‐smoker) identity (Johnson et al., 2003; Meijer et al., 2017; Vangeli & West, 2012). However, this transition is rarely abrupt. Smoker and ex‐smoker identities often coexist during cessation, with smoker identity gradually weakening as ex‐smoker identity becomes more salient (Vangeli et al., 2010; Vangeli & West, 2012). This process may nevertheless destabilize individuals' sense of self, giving rise to perceived self‐discontinuity. The present research examined whether this subjective disruption contributes to smoking relapse.

### Self‐discontinuity and smoking relapse

Self‐continuity refers to the perception that one's self‐concept remains coherent and stable over time despite changes in life circumstances. In contrast, self‐discontinuity reflects a perceived rupture in personal identity, such that individuals experience their current self as disconnected from their past self. It is characterized by a sense of temporal instability and is associated with feelings of confusion, disconnection, and identity loss (Chandler & Proulx, 2008; Iyer & Jetten, 2011).

A large body of research indicates that maintaining a sense of self‐continuity is a fundamental psychological motive (Breakwell, 1986; Vignoles et al., 2006), closely linked to well‐being and self‐esteem (Sani, 2008). Despite life changes, individuals are motivated to preserve a stable sense of self and to avoid experiences of discontinuity. Nevertheless, major life transitions, such as smoking cessation, may disrupt this continuity and create a sense of temporal rupture. When such disruptions occur, individuals often engage in behaviors aimed at restoring a continuous self‐narrative (Rutchick et al., 2018; Zhao et al., 2022), typically by aligning their current actions with their past self (Kim et al., 2017; Kim & Wohl, 2015; Wohl et al., 2018). For instance, Wohl et al. (2018) showed that individuals with severe gambling or alcohol problems reduced their addictive behaviors after reflecting on how these behaviors disrupted their former non‐addicted self, thereby restoring a sense of self‐continuity.

Smoking cessation represents a context in which self‐continuity may be particularly vulnerable. Qualitative research indicates that individuals who quit smoking often report experiences of identity disruption, including feelings of disconnection, a sense of identity loss, and residual attraction to a former smoker identity (Dijkstra & Borland, 2003; Meijer et al., 2020; Vangeli et al., 2010; Vangeli & West, 2012). Notley and Colllins (2018), for instance, found that ex‐smokers often describe quitting as a rupture in their personal identity narrative, with relapse serving to reconnect with a lost or missed identity. Together, these findings suggest that smoking cessation is a major life transition that may undermine self‐continuity and thereby increase vulnerability to relapse. However, to date, no quantitative research has directly examined the role of self‐discontinuity in smoking relapse. The present study addressed this gap.

Importantly, self‐discontinuity is conceptually related to, yet distinct from, constructs such as identity conflict, identity instability, or self‐concept clarity. Whereas these constructs primarily refer to the coherence or compatibility among one's multiple identities at a given point in time, self‐discontinuity specifically refers to the extent to which individuals experience a sense of coherence and stability in their self across time. This temporal perspective is particularly relevant to smoking cessation, which unfolds as a long‐term and often cyclical process involving repeated quit attempts, periods of abstinence, and potential relapses. By focusing on how individuals perceive the continuity of their sense of self across their personal life trajectory, self‐discontinuity complements and extends existing identity‐based models that have predominantly emphasized the salience of smoker and ex‐smoker identities (Hertel & Mermelstein, 2016; Meijer et al., 2017, 2018). Most fundamentally, it provides additional explanatory value by capturing a temporal dimension of identity processes that has received little attention in smoking research.

### Smoker and ex‐smoker identities as mediating pathways

Smoking relapse is strongly shaped by smoker and ex‐smoker identities. Prior research indicates that relapse risk increases when smoking cessation is not supported by a full identity transition, namely, when individuals continue to endorse a smoker identity and fail to adequately adopt an ex‐smoker (or non‐smoker) identity (e.g. Meijer et al., 2018). For instance, Callaghan et al. (2021) showed that former smokers who continue to identify as “smokers” were more likely to relapse within the following year than those who identified as “smokers trying to quit,” “smokers who have chosen to no longer smoke,” “ex‐smokers,” or “non‐smokers.” Similarly, Falomir‐Pichastor et al. (2020) found that dependent ex‐smokers are more likely to fail to maintain abstinence when their self‐concept contained a greater proportion of smoker identity relative to ex‐smoker identity. Although these findings establish the central role of identity in relapse, the mechanisms through which smoker and ex‐smoker identities are maintained or transformed after cessation remain unclear.

We propose that smoking‐related self‐discontinuity may hinder the identity reconfiguration necessary for successful cessation. Specifically, self‐discontinuity may reinforce the persistence of smoker identity while inhibiting the adoption of an ex‐smoker identity. When ex‐smokers experience a disruption in the continuity of the self over time, they may attempt to restore continuity by maintaining a smoker identity, namely, a familiar and continuity‐preserving aspect of the self, while resisting the internalization of an ex‐smoker identity, which may be experienced as psychologically destabilizing. This process, in turn, may increase the likelihood of relapse. Accordingly, we examined smoker and ex‐smoker identities as mediating mechanisms linking self‐discontinuity to relapse risk. We hypothesized indirect effects of self‐discontinuity on relapse through increases in smoker identity and decreases in ex‐smoker identity.

This account is consistent with recent work by Blondé and Falomir‐Pichastor (2026), which showed that, among smokers, perceived self‐discontinuity, defined as the belief that quitting smoking would disrupt one's sense of self, indirectly reduced engagement in quit attempts over time by reinforcing smoker identity and inhibiting the adoption of an ex‐smoker identity. In addition, two experimental studies demonstrated that enhancing perceived self‐continuity (i.e. reassuring individuals that quitting would not disrupt their sense of self) increased the salience of ex‐smoker identity (relative to smoker identity) and indirectly promoted cessation‐related behaviors. The present study extended this research to a sample of ex‐smokers and focused specifically on smoking relapse.

Within this framework, self‐discontinuity is conceptualized as a broader meta‐identity belief concerning the perceived temporal continuity of the self, whereas smoker and ex‐smoker identities represent specific self‐contents. Self‐discontinuity is therefore theorized as an overarching perception of self‐structure that may shape identity reconfiguration during smoking cessation. However, the temporal direction of this relationship remains empirically uncertain and is examined in the present study.

### The present research

The present study examined longitudinal associations among self‐discontinuity, smoker and ex‐smoker identity, and smoking relapse. To test the proposed mediation model, we conducted a 12‐month longitudinal study of a large cohort of ex‐smokers assessed at four time points, each separated by 4‐month intervals. Self‐discontinuity, smoker identity, and ex‐smoker identity were measured at each wave, whereas smoking relapse was assessed from the second wave onward.

Multilevel analyses were conducted to examine within‐person associations rather than between‐person differences. Within‐person longitudinal analyses are well suited to capturing change over time because they distinguish dynamic intraindividual fluctuations from stable interindividual differences. Accordingly, the study tested whether within‐person changes in self‐discontinuity were concurrently and prospectively associated with changes in smoker and ex‐smoker identity and, in turn, with relapse risk. Sensitivity analyses further examined the directionality of the proposed mediation model by reversing the temporal ordering of the predictor and mediators (see Fiedler et al., 2018).

The study received ethical approval from the institutional review board of our institution (No. CUREG‐2021‐10‐114). Informed consent was obtained from all participants prior to their participation in the study, with consent indicated by proceeding with the study after being informed about the study and its conditions of participation. The study was not preregistered but closely followed the preregistered hypotheses and analytic procedures reported in Blondé and Falomir‐Pichastor (2026; see Study 2). The dataset, analysis scripts, and the accompanying codebook are available at https://osf.io/3gwu4.

## METHOD

### Participants and procedure

Participants were recruited through the online platform Prolific. Eligibility was determined using Prolific's prescreening filters. Participants were required to self‐identify as ex‐smokers at the time of participation. In addition, eligibility was restricted to individuals residing in the United Kingdom or the United States, as these constitute the largest English‐speaking participant pools on Prolific.

To account for expected attrition across the longitudinal design, we aimed to recruit a large baseline sample of more than 1000 ex‐smokers. Accordingly, 1025 participants who reported being ex‐smokers at the time of the baseline survey were initially recruited. Upon closer inspection of the baseline data, it became apparent that a substantial proportion of participants had quit smoking many years prior to study entry (an average of 8.78 years). For example, 28.70% reported being abstinent for more than 10 years. While such individuals can reliably be classified as ex‐smokers, extended abstinence may be associated with a low probability of relapse and reduced salience of identity‐related processes, thereby limiting our ability to observe meaningful change over time. Indeed, perceptions of self‐discontinuity and smoker identity may be substantially diminished after many years of abstinence. Including individuals who have been abstinent for many years may therefore attenuate associations of interest and bias estimates toward null effects. Accordingly, we restricted our analyses to participants who had quit smoking within the 5 years preceding the baseline assessment. This decision was guided by empirical evidence provided by García‐Rodríguez et al. (2013), showing that the risk of relapse remains relatively high during the first years following cessation (exceeding 50% within the first year and decreasing to approximately 25% after 5 years of abstinence), after which the decline in relapse risk becomes markedly slower and stabilizes at much lower levels. This criterion allowed us to focus on a population for whom identity‐related processes and relapse‐relevant dynamics are expected to be most operative, while retaining a sufficiently large sample for longitudinal analyses. In a similar vein, the Betty Ford Institute Consensus Panel proposed that sustained recovery may be considered achieved after five or more years of continuous abstinence (Betty Ford Institute Consensus Panel, 2007).

After excluding participants who had quit smoking more than 5 years prior to the baseline assessment (*n* = 467), those who failed an attention check (*n* = 4), and those who reported not being ex‐smokers at baseline despite indicating otherwise during initial screening (*n* = 111), the final sample consisted of 443 adult ex‐smokers (218 women), aged between 18 and 76 years (M = 39.91, *SD* = 11.87). Most participants resided in the United Kingdom. Over the study period, participant retention was relatively high, with 337 participants (76.1% retention) completing the survey at Time 2, 316 (71.3%) at Time 3, and 283 (63.9%) at Time 4 (a flow diagram is provided in Figure S1). Participants were compensated for each survey wave, with payments increasing over the course of the study. Before quitting, participants smoked an average of 13.56 cigarettes per day (*SD* = 8.49). At baseline, they reported that they had been abstinent for an average of 26.32 months (*SD* = 18.22; approximately 2.19 years) and had smoked for an average of 15.61 years (*SD* = 10.73) prior to quitting. Additional demographic characteristics of the sample are provided in Table S1.

The study followed a 12‐month longitudinal design with four waves separated by 4‐month intervals. At baseline, participants completed measures of self‐discontinuity, smoker and ex‐smoker identity, demographic information (e.g. age and gender), and smoking history (see the Measures section).^1^ At each follow‐up, participants were recontacted via Prolific and invited to complete the survey again. In these follow‐ups, they reported their current smoking status (see smoking relapse measure). Self‐discontinuity, smoker identity, and ex‐smoker identity were assessed at each wave, whereas smoking relapse was assessed from the second wave onward. Each survey required approximately 10 min to complete.

### Measures

#### Self‐discontinuity

Self‐discontinuity was assessed using an adapted version of the five‐item scale developed by Blondé and Falomir‐Pichastor (2026), tailored for use with a population of ex‐smokers. The items captured the extent to which quitting smoking is experienced as a rupture in personal identity (e.g. “Since I quit smoking, I feel like I am no longer the same,” “Now that I have quit smoking, I am no longer who I have always been,” and “Since I have quit smoking, I feel disconnected from who I am”). Responses were provided on a 7‐point Likert‐type scale (1 = *not at all*, 7 = *very much*), with higher scores indicating greater self‐discontinuity. The scale demonstrated excellent internal consistency at each measurement occasion (T1: M = 2.95, *SD* = 1.41, *α* = .85; T2: M = 2.65, *SD* = 1.34, *α* = .84; T3: M = 2.54, *SD* = 1.30, *α* = .85; T4: M = 2.53, *SD* = 1.35, *α* = .87).^2^

#### Smoker and ex‐smoker identity

We assessed smoker and ex‐smoker identity at each time point using items already used in previous research (e.g. Blondé & Falomir‐Pichastor, 2026). Participants rated three items measuring smoker identity (e.g. “Do you see yourself as a smoker?”; T1: M = 2.51, *SD* = 1.29, *α* = .71; T2: M = 2.39, *SD* = 1.32, *α* = .76; T3: M = 2.39, *SD* = 1.26, *α* = .73; T4: M = 2.47, *SD* = 1.32, *α* = .71) and three items measuring ex‐smoker identity (e.g. “Do you see yourself as an ex‐smoker?”; T1: M = 4.62, *SD* = 1.42, *α* = .66; T2: M = 4.51, *SD* = 1.53, *α* = .73; T3: M = 4.46, *SD* = 1.47, *α* = .71; T4: M = 4.41, *SD* = 1.63, *α* = .79). All items were rated on a 7‐point scale (1 = *no, not at all*, 7 = *yes, absolutely*).

#### Smoking relapse

Relapse was assessed at three follow‐ups (Waves 2, 3, and 4). At each wave, participants indicated their smoking status by selecting one of six options: (1) “I used to smoke at the time of the last questionnaire, and I have not attempted to quit since then,” (2) “I used to smoke at the time of the last questionnaire, I have attempted to quit since then, but I am currently smoking again,” (3) “I used to smoke at the time of the last questionnaire, I have attempted to quit since then, and I am still currently not smoking,” (4) “I did not smoke at the time of the last questionnaire, I have smoked cigarettes again since then, but I am not currently smoking anymore,” (5) “I did not smoke at the time of the last questionnaire, I have smoked cigarettes again since then, and I am still currently smoking,” and (6) “I did not smoke at the time of the last questionnaire and I have not smoked a single cigarette since then.” Participants were explicitly instructed that a relapse or quit attempt must have lasted at least 24 h. From Waves 2 to 4, we created relapse indicators that operationalized relapse as any smoking during the interval between two consecutive assessments. Specifically, participants were classified as having experienced a relapse if they reported having smoked cigarettes at any point since the previous assessment, which included participants who had been smoking at the previous assessment and remained smoking, as well as those who had made a quit attempt. Accordingly, at Wave 2, Responses 4 and 5 were coded as relapse (1), whereas Response 6 was coded as no relapse (0). At Waves 3 and 4, Responses 1–5 were coded as relapse (1), whereas Response 6 was coded as no relapse (0). This inclusive definition was adopted to ensure sufficient relapse events and within‐person variation across waves and individuals to support the planned longitudinal analyses.^3^ At Wave 2, 94 participants (21.2%) were classified as having experienced a relapse; at Wave 3, 104 participants (23.5%); and at Wave 4, 103 participants (23.3%). A detailed breakdown of the six response options and their frequencies at each wave is provided in Table S2.

#### Covariates

At baseline, we measured participants' past smoking consumption (i.e. the average number of cigarettes smoked per day), duration of the current quit attempt (i.e. the number of months since quitting), and duration prior to the quit attempt (i.e. the number of months of smoking before quitting).

### Construction of time‐structured variables

Rather than relying on raw wave‐specific values (e.g. T1 self‐discontinuity and T2 smoker identity), we used time‐structured variables. This approach allowed temporal ordering to be clearly specified and, when implemented within a multilevel framework, to isolate within‐person fluctuations across waves. Three types of time‐structured variables were distinguished. First, contemporaneous variables were created for self‐discontinuity, smoker identity, ex‐smoker identity, and smoking relapse. These variables captured participants' current levels of each construct at a given measurement occasion (i.e. at time *t*) and reflected within‐person variation around individuals' typical levels. Second, a prospective variable was constructed for smoking relapse to represent the outcome at the subsequent measurement occasion (*t* + 1). This variable captured relapse status at the wave immediately following the current assessment and served as the primary outcome in the examined models. Third, lagged variables were created for all time‐varying variables (i.e. self‐discontinuity, smoker identity, ex‐smoker identity, and relapse) to represent their values at the immediately preceding wave (*t* − 1). Lagged variables were set to missing at the first measurement occasion for each participant, as no prior observation was available at baseline.

### Missing data

Missing data were handled using multiple imputation by chained equations (MICE), generating 15 imputed datasets. Imputation was applied only to contemporaneous variables; lagged (*t* − 1) and prospective (*t* + 1) variables were not imputed directly but were derived from the imputed contemporaneous variables. This approach preserved temporal dependencies while avoiding the imputation of derived variables. Continuous variables (self‐discontinuity, smoker identity, and ex‐smoker identity) were imputed using linear regression models, whereas smoking relapse, a binary outcome, was imputed using logistic regression. No additional auxiliary variables were included in the imputation models. The proportion of missing observations varied across variables and assessment waves, ranging from 0% at baseline to 36.1% at Wave 4. Missingness generally increased across waves, with variable‐specific rates reported in Table S3. Percentages were calculated relative to the baseline sample (*N* = 443).

### Analytic strategy

Two complementary longitudinal mediation models were tested using multilevel modeling in Mplus. Both models distinguished within‐person from between‐person effects to separate dynamic processes unfolding within individuals over time from stable differences between individuals. This distinction is essential because associations observed at the between‐person level do not necessarily reflect within‐individual processes across time, and conflating these levels can lead to misleading inferences about psychological change (Curran & Bauer, 2011; Hamaker et al., 2015).

The first model was partially prospective (Model 1). Contemporaneous effects were estimated to test whether within‐person changes (i.e. fluctuations relative to the individual's typical level) in self‐discontinuity at a given wave (*t*) predicted subsequent smoking relapse at the next wave (*t* + 1, lead variable) via within‐person changes in smoker identity and ex‐smoker identity measured at the same wave (*t*). This model combined a prospective outcome with mediators assessed concurrently with the predictor. The autoregressive effect of smoking relapse was included to account for temporal stability. At the between‐person level, the model included only time‐invariant covariates related to smoking history (i.e. past tobacco consumption, duration of prior smoking, and duration of the quit attempt).

The second multilevel model was fully prospective (Model 2). Lagged within‐person predictors measured at the previous wave (*t* − 1) were used to predict contemporaneous mediators (*t*), which in turn predicted subsequent smoking relapse (*t* + 1, lead variable). This model provided a complete temporal separation between predictors, mediators, and outcomes, allowing a fully prospective test of within‐person mediation. Specifically, we tested whether within‐person deviations in self‐discontinuity at the previous wave (*t* − 1) predicted changes in smoker identity and ex‐smoker identity at the current wave (*t*), which subsequently influenced relapse at the next wave (*t* + 1). To isolate dynamic within‐person change and avoid conflating prospective associations with temporal stability, autoregressive paths were included for self‐discontinuity, smoker identity, ex‐smoker identity, and relapse. As in the first model, residual correlations between smoker identity and ex‐smoker identity were freely estimated, and time‐invariant covariates were included as between‐person predictors of relapse.

In both models, within‐person indirect effects were estimated using the product‐of‐coefficients method, with total effects calculated as the sum of direct and indirect paths. Self‐discontinuity, smoker identity, and ex‐smoker identity were treated as continuous variables, whereas smoking relapse was modeled as a categorical outcome. Models were estimated using the weighted least squares estimator with mean and variance adjustment (WLSMV), appropriate for two‐level models including categorical outcomes (Brown, 2015).

## RESULTS

Results from Model 1 are presented in Table 1, and those from Model 2 are in Table 2. The sensitivity analyses are reported in Table 3.

**TABLE 1:** Results of the partially prospective mediation model (Model 1).

	*β* (*SE*)	*p*	95% CI
Within‐person			
Auto‐regressive path			
Relapse	.267 (.10)	.006	[.077, .457]
Direct associations			
Self‐discontinuity (*t*) → Smoker identity (*t*)	.269 (.02)	<.001	[.233, .305]
Self‐discontinuity (*t*) → Ex‐smoker identity (*t*)	.092 (.02)	<.001	[.051, .134]
Self‐discontinuity (*t*) → Relapse (*t* + 1)	−.027 (.09)	.758	[−.199, .145]
Smoker identity (*t*) → Relapse (*t* + 1)	.213 (.08)	.006	[.061, .364]
Ex‐smoker identity (*t*) → Relapse (*t* + 1)	.080 (.08)	.302	[−.072, .231]
Indirect effects			
Self‐discontinuity (*t*) → Smoker identity (*t*) → Relapse (*t* + 1)	.044 (.02)	.008	[.012, .077]
Self‐discontinuity (*t*) → Ex‐smoker identity (*t*) → Relapse (*t* + 1)	.006 (.01)	.322	[−.006, .017]
Total indirect effect	.050 (.02)	.005	[.015, .085]
Between‐person			
Quit attempt duration	−.401 (.06)	<.001	[−.524, −.277]
Smoking duration	−.185 (.07)	.013	[−.329, −.040]
Past smoking consumption	−.103 (.07)	.128	[−.236, .030]

*Note*: Coefficients represent standardized within‐person estimates from a two‐level model. The model is partially prospective, with contemporaneous predictors and mediators measured at time *t* and smoking relapse measured at the subsequent wave (*t* + 1).

Abbreviation: CI, confidence interval.

**TABLE 2:** Results of the fully prospective mediation model (Model 2).

	*β* (*SE*)	*p*	95% CI
Within‐person			
Auto‐regressive paths			
Self‐discontinuity	.535 (.02)	<.001	[.493, .578]
Smoker identity	.705 (.02)	<.001	[.673, .737]
Ex‐smoker identity	.622 (.02)	<.001	[.588, .656]
Relapse	.412 (.05)	<.001	[.316, .508]
Direct associations			
Self‐discontinuity (*t* − 1) → Smoker identity (*t*)	−.001 (.03)	.956	[−.052, .049]
Self‐discontinuity (*t* − 1) → Ex‐smoker identity (*t*)	−.024 (.03)	.395	[−.078, .031]
Self‐discontinuity (*t* − 1) → Relapse (*t* + 1)	.021 (.18)	.905	[−.325, .367]
Smoker identity (*t*) → Relapse (*t* + 1)	.343 (.10)	.001	[.150, .537]
Ex‐smoker identity (*t*) → Relapse (*t* + 1)	.043 (.12)	.710	[−.183, .269]
Indirect effects			
Self‐discontinuity (*t* − 1) → Smoker identity (*t*) → Relapse (*t* + 1)	.001 (.01)	.952	[−.014, .013]
Self‐discontinuity (*t* − 1) → Ex‐smoker identity (*t*) → Relapse (*t* + 1)	−.001 (.01)	.744	[−.005, .004]
Total indirect effect	−.001 (.01)	.869	[−.015, .013]
Between‐person			
Quit attempt duration	−.374 (.07)	<.001	[−.497, −.251]
Smoking duration	−.148 (.07)	.047	[−.293, −.002]
Past smoking consumption	−.127 (.06)	.042	[−.249, −.005]

*Note*: Coefficients represent standardized within‐person estimates from a two‐level model. The model is fully prospective, with lagged predictors measured at the previous wave (*t* − 1), mediators measured at the current wave (*t*), and smoking relapse measured at the subsequent wave (*t* + 1).

Abbreviation: CI, confidence interval.

**TABLE 3:** Results of sensitivity analyses.

	*β* (*SE*)	*p*	95% CI
Model 1			
Within‐person			
Auto‐regressive path			
Relapse	.265 (.10)	.007	[.074, .456]
Direct associations			
Smoker identity (*t*) → Self‐discontinuity (*t*)	.269 (.02)	<.001	[.234, .304]
Ex‐smoker identity (*t*) → Self‐discontinuity (*t*)	.093 (.02)	<.001	[.053, .132]
Smoker identity (*t*) → Relapse (*t* + 1)	.214 (.08)	.007	[.057, .370]
Ex‐smoker identity (*t*) → Relapse (*t* + 1)	.081 (.08)	.292	[−.070, .233]
Self‐discontinuity (*t*) → Relapse (*t* + 1)	−.029 (.08)	.729	[−.191, .134]
Indirect effects			
Smoker identity (*t*) → Self‐discontinuity (*t*) → Relapse (*t* + 1)	−.007 (.02)	.731	[−.045, .031]
Ex‐smoker identity (*t*) → Self‐discontinuity (*t*) → Relapse (*t* + 1)	−.002 (.01)	.720	[−.013, .009]
Total indirect effect	−.009 (.03)	.728	[−.057, .040]
Between‐person			
Quit attempt duration	−.402 (.06)	<.001	[−.525, −.279]
Smoking duration	−.187 (.07)	.012	[−.331, −.042]
Past smoking consumption	−.098 (.07)	.142	[−.229, .033]
Model 2			
Within‐person			
Auto‐regressive paths			
Self‐discontinuity	.553 (.02)	<.001	[.512, .593]
Smoker identity	.703 (.01)	<.001	[.676, .731]
Ex‐smoker identity	.618 (.02)	<.001	[.584, .652]
Relapse	.433 (.05)	<.001	[.328, .538]
Direct associations			
Smoker identity (*t* − 1) → Self‐discontinuity (*t*)	.132 (.02)	<.001	[.090, .173]
Ex‐smoker identity (*t* − 1) → Self‐discontinuity (*t*)	.079 (.02)	.001	[.032, .125]
Smoker identity (*t* − 1) → Relapse (*t* + 1)	.252 (.13)	.047	[.003, .502]
Ex‐smoker identity (*t* − 1) → Relapse (*t* + 1)	.079 (.14)	.574	[−.197, .355]
Self‐discontinuity (*t*) → Relapse (*t* + 1)	.097 (.13)	.468	[−.164, .357]
Indirect effects			
Smoker identity (*t* − 1) → Self‐discontinuity (*t*) → Relapse (*t* + 1)	.033 (.02)	.078	[−.004, .070]
Ex‐smoker identity (*t* − 1) → Self‐discontinuity (*t*) → Relapse (*t* + 1)	.004 (.01)	.589	[−.011, .019]
Total indirect effect	.038 (.02)	.086	[−.005, .080]
Between‐person			
Quit attempt duration	−.360 (.06)	<.001	[−.476, −.245]
Smoking duration	−.131 (.07)	.051	[−.264, .001]
Past smoking consumption	−.131 (.06)	.029	[−.249, −.013]

*Note*: Coefficients represent standardized within‐person estimates from a two‐level model.

Abbreviation: CI, confidence interval.

### Model 1: Partial temporal ordering (*t* → *t* → *t* + 1)

#### Within‐person effects

Within‐person analyses revealed concurrent positive associations between self‐discontinuity and smoker identity (*β* = .269, *p* < .001), as well as between self‐discontinuity and ex‐smoker identity (*β* = .092, *p* < .001). In turn, smoker identity was associated with an increased likelihood of relapse at the subsequent assessment (*β* = .213, *p* = .006), whereas ex‐smoker identity was not significantly related to relapse risk (*β* = .080, *p* = .302). The direct within‐person association between self‐discontinuity and relapse was not significant (*β* = −.027, *p* = .758), nor was the total effect of self‐discontinuity on relapse (*β* = .029, *p* = .649). As expected, relapse at the previous wave predicted relapse at the subsequent wave (*β* = .267, *p* = .006).

**FIGURE 1:**
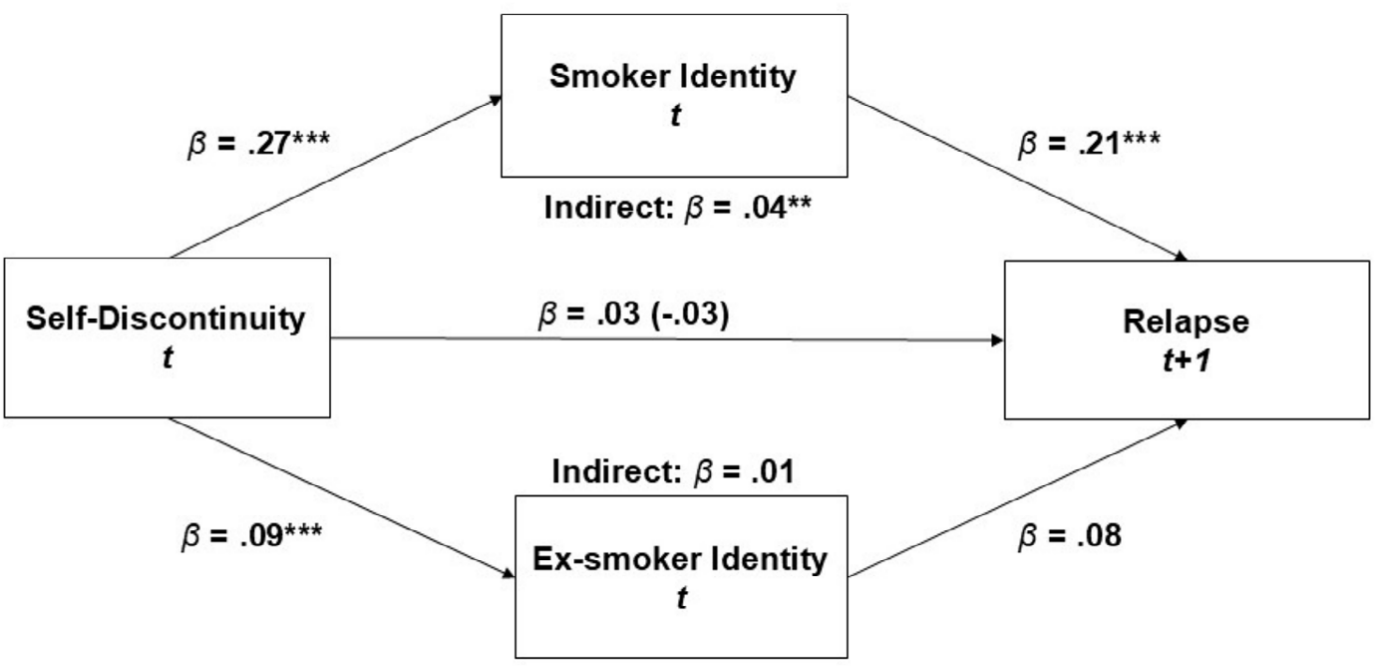
Partially prospective within‐person mediation model predicting subsequent smoking relapse (all estimates are standardized). *Note*: The model depicts within‐person associations linking self‐discontinuity at a given measurement occasion (*t*) to smoking relapse at the subsequent occasion (*t* + 1) via contemporaneous smoker identity and ex‐smoker identity (*t*). All variables represent within‐person deviations across waves. Autoregressive paths and between‐person covariates are omitted from the figure for clarity. **p < .01, ***p < .001.

There was a significant indirect effect of self‐discontinuity on relapse through smoker identity (*β* = .044, *p* = .008), indicating that within‐person periods of heightened self‐discontinuity were associated with an increased likelihood of subsequent relapse via temporarily stronger smoker identity (see Figure 1). In contrast, the indirect effect via ex‐smoker identity was not significant (*β* = .006, *p* = .322). The total indirect effect, reflecting the combined contribution of both identity pathways, was significant (*β* = .050, *p* = .005). Together, these findings indicated that within‐person fluctuations in self‐discontinuity are linked to relapse risk primarily through their contemporaneous association with smoker identity rather than ex‐smoker identity.

#### Between‐person effects

At the between‐person level, relapse risk was significantly associated with quit attempt duration and smoking duration. Individuals who had quit more recently (*β* = −.401, *p* < .001) and those with a shorter smoking history (*β* = −.185, *p* = .013) were more likely to relapse across the study period. In contrast, past tobacco consumption was not significantly related to relapse risk (*β* = −.103, *p* = .128).

### Model 2: Full temporal ordering (*t* − 1 → *t* → *t* + 1)

#### Within‐person effects

Autoregressive paths indicated substantial temporal stability for all constructs across consecutive waves. Self‐discontinuity (*β* = .535, *p* < .001), smoker identity (*β* = .705, *p* < .001), ex‐smoker identity (*β* = .622, *p* < .001), and relapse (*β* = .412, *p* < .001) were each strongly and positively predicted by their respective values at the previous measurement occasion. In contrast to the previous model, the prospective within‐person analyses provided no evidence that earlier increases in self‐discontinuity predicted subsequent changes in identity‐related variables. Lagged within‐person deviations in self‐discontinuity were not associated with later smoker identity (*β* = −.001, *p* = .956) or ex‐smoker identity (*β* = −.024, *p* = .395). Smoker identity at the previous wave was a significant predictor of relapse at the subsequent wave (*β* = .343, *p* = .001), whereas higher ex‐smoker identity was not associated with relapse risk (*β* = .043, *p* = .710). The lagged direct effect of self‐discontinuity on relapse was not significant (*β* = .021, *p* = .905), nor was the total effect of self‐discontinuity on relapse (*β* = .015, *p* = .912). Consistent with these findings, no prospective within‐person mediation effects were observed. Indirect effects of self‐discontinuity on relapse via smoker identity (*β* = .001, *p* = .952) and ex‐smoker identity (*β* = −.001, *p* = .744) were not significant, and the total indirect effect was likewise non‐significant (*β* = −.001, *p* = .869).

#### Between‐person effects

At the between‐person level, relapse was associated with quit attempt duration (*β* = −.374, *p* < .001), smoking duration (*β* = −.148, *p* = .047), and past tobacco consumption (*β* = −.127, *p* = .042).

### Sensitivity analyses

To assess the robustness and directionality of the proposed mediation model, we re‐estimated Models 1 and 2 with the mediation structure reversed, specifying smoker and ex‐smoker identities (at *t* and *t* − 1) as predictors and self‐discontinuity (at *t*) as the mediator. Unlike the original models, the reversed Model 1 showed no significant indirect effect of smoker identity on relapse through self‐discontinuity (*β* = −.007, *p* = .731). In the reversed Model 2, both smoker identity (*β* = .132, *p* < .001) and ex‐smoker identity (*β* = .079, *p* < .001) were significantly associated with subsequent changes in self‐discontinuity, but indirect effects of smoker identity (*β* = .033, *p* = .078) and ex‐smoker identity (*β* = .004, *p* = .589) on relapse via self‐discontinuity were both non‐significant.

In addition, all primary analyses were repeated while controlling for age, gender, and socioeconomic status (income and education). The main within‐person associations remained unchanged after adjustment. However, the association between smoking duration and relapse was attenuated and became non‐significant after controlling for these covariates. Given the strong relationship between smoking duration and age, this finding suggested that the observed association between smoking duration and relapse should be interpreted cautiously and may not reflect an independent effect of smoking duration. These results are reported in Tables S4 and S5. Similarly, we conducted complete‐case sensitivity analyses. For Model 1, associations between self‐discontinuity and both identity measures remained similar to the primary analyses, although the indirect effects through smoker and ex‐smoker identity were no longer significant. Detailed results are reported in Table S7. Model 2 could not be reliably estimated because the substantial loss of observations due to missing data resulted in a singular information/weight matrix under WLSMV.

## DISCUSSION

The present research examined the role of identity‐related mechanisms in understanding smoking relapse among former smokers who had quit within the previous 5 years. Multilevel analyses were conducted to test a longitudinal mediation model capturing within‐person associations among perceived self‐discontinuity, smoker and ex‐smoker identity, and smoking relapse over time, while controlling for stable between‐person differences related to smoking history (i.e. past tobacco consumption, quit attempt duration, and smoking duration).

Analyses of Model 1 revealed a positive within‐person indirect effect of self‐discontinuity on subsequent relapse through smoker identity, but not through ex‐smoker identity. This indicates that periods during which individuals experienced higher‐than‐usual levels of self‐discontinuity (relative to their own average across waves) were concurrently and positively associated with smoker identity, which in turn predicted a higher likelihood of relapse at the following assessment. Although this indirect effect was modest in magnitude, as is common for indirect effects involving multiple regression paths, it does not, in itself, establish a causal mechanism. Nevertheless, it provides evidence for a psychological process whereby self‐discontinuity may increase subsequent relapse risk through its concurrent association with smoker identity. Importantly, sensitivity analyses testing an alternative mediation model in which the predictor and mediators were reversed showed no significant indirect effect through self‐discontinuity. Moreover, this indirect effect emerged in the absence of a significant direct effect of self‐discontinuity on relapse, indicating that perceived self‐discontinuity does not independently increase relapse risk but is associated with relapse only indirectly through its concurrent association with smoker identity.

By contrast, analyses of Model 2 indicated that lagged deviations in self‐discontinuity did not predict subsequent changes in either smoker or ex‐smoker identity and accordingly yielded no indirect effects on relapse. This pattern suggests that the relationship between self‐discontinuity and smoker identity is temporally proximal rather than delayed, reflecting a concurrent rather than lagged effect of self‐discontinuity on identity change. From this perspective, self‐discontinuity may be better understood as a situational correlate of fluctuations in smoker identity rather than as a stable force driving long‐term identity transformation. Consequently, the indirect effect observed in the partially prospective model should be interpreted as reflecting temporally proximal associations rather than evidence of a fully longitudinal mediation process. While self‐discontinuity, smoker identity, and relapse are dynamically linked, the present data did not support a robust longitudinal mediation pathway and did not support our original prediction that higher self‐discontinuity would weaken ex‐smoker identity over time.

Although relatively small in magnitude, sensitivity analyses further showed that lagged levels of both smoker and ex‐smoker identities predicted subsequent increases in self‐discontinuity, whereas lagged self‐discontinuity did not predict later changes in either identity (see Model 2). This asymmetry suggests that changes in smoker and ex‐smoker identity are more likely to precede perceptions of self‐discontinuity than the reverse. Accordingly, these findings did not support our original hypothesis that higher self‐discontinuity would be an antecedent of changes in smoker and ex‐smoker identity. Self‐discontinuity may therefore be more appropriately conceptualized as an emerging consequence of identity reconfiguration during smoking cessation rather than its primary temporal antecedent. Rather than initiating changes in smoker and ex‐smoker identities, perceptions of self‐discontinuity may arise as individuals negotiate the transition between these identities and attempt to integrate competing self‐definitions into a coherent sense of self. This disruption in perceived temporal self‐continuity may specifically emerge during the process of integrating a former smoker identity with an emerging ex‐smoker identity, rather than reflecting a strictly linear process of identity change. Consistently, both smoker and ex‐smoker identities were positively associated with self‐discontinuity, suggesting that feelings of discontinuity may be particularly likely when a salient smoker identity persists alongside the development of an ex‐smoker identity (e.g. Vangeli et al., 2010; Vangeli & West, 2012). Importantly, this interpretation does not diminish the relevance of self‐discontinuity in smoking cessation. Although it may emerge downstream of identity change, our findings suggest that it remains involved in relapse processes through its associations with smoker identity and subsequent relapse risk in the partially prospective model. Thus, self‐discontinuity may be understood as a contextual marker of identity reorganization during smoking cessation, closely intertwined with identity processes relevant to relapse vulnerability.

An important finding was the absence of both a direct effect of ex‐smoker identity on relapse risk and an indirect effect through ex‐smoker identity in any of the tested models. This pattern of results did not support our original hypothesis that self‐discontinuity would be negatively associated with ex‐smoker identity. This was unexpected given previous research showing that ex‐smoker identity predicts smoking cessation and relapse outcomes (Callaghan et al., 2021; Falomir‐Pichastor et al., 2020; Meijer et al., 2018). Several explanations may account for this discrepancy. First, the reliability of the ex‐smoker identity scale was relatively modest at baseline (*α* = .66), which may have attenuated its predictive power. Second, as we mentioned above, smoker and ex‐smoker identities are not simply opposite ends of a single continuum but may coexist during the cessation process. Individuals may progressively adopt an ex‐smoker identity while continuing to maintain a residual smoker identity (Vangeli et al., 2010; Vangeli & West, 2012). Consequently, self‐discontinuity may reinforce the persistence of smoker identity without necessarily weakening ex‐smoker identity. In addition, relapse may be more directly driven by the continued salience of smoker identity than by the absence of an ex‐smoker identity, as smoker identity is more closely aligned with the act of smoking itself. Nevertheless, these findings should not be interpreted as evidence that ex‐smoker identity is unimportant. Rather, they suggest that its role in smoking cessation may be more complex than originally hypothesized and warrants further investigation.

An important contribution of the present study lies in its focus on within‐person processes. Rather than asking whether individuals who generally experience greater self‐discontinuity are more likely to relapse than others, our analyses examined whether increases in self‐discontinuity relative to an individual's own usual level were associated with subsequent relapse risk. The findings therefore suggested that relapse vulnerability is better understood as a dynamic process that fluctuates within individuals over time rather than as a consequence of stable individual differences. This perspective has important implications for both theory and intervention, as it suggests that changes in self‐discontinuity and smoker identity may represent time‐varying psychological risk factors that could be monitored and targeted during smoking cessation.

More broadly, the present research underscored the value of adopting an identity‐based perspective to understand smoking‐related behaviors, particularly relapse processes. Although prior work has primarily focused on how smoker and ex‐smoker identities shape quit attempts and relapse risk (Callaghan et al., 2021; Falomir‐Pichastor et al., 2020), our findings pointed to the importance of considering broader meta‐identity concerns, such as self‐continuity. Considering how smokers and ex‐smokers personally define themselves is necessary, but not sufficient. It is equally important to understand how engaging in an identity transition, which involves relinquishing a self‐defining identity and adopting a new, potentially destabilizing one, is perceived to affect the coherence of the broader self‐system. Accordingly, we encourage future research to further examine meta‐identity factors and processes that shape identity transitions during smoking cessation and other forms of behavioral change.

The present findings also have practical implications for cessation interventions. Although the current study did not establish causal effects, it suggests that increases in self‐discontinuity and smoker identity may signal periods of heightened vulnerability to relapse. Smoking cessation programs could therefore benefit from paying greater attention to how individuals experience changes in their sense of self during the quitting process, rather than focusing exclusively on smoking behavior or craving. Interventions that support the integration of smoking cessation into a continuous sense of self, while reducing the salience of smoker identity, may facilitate long‐term abstinence. More broadly, assessing identity‐related experiences throughout the cessation process may help identify individuals at increased risk of relapse and guide the timing of additional psychological support.

### Limitations and future directions

Several limitations should be acknowledged. First, the present study focused specifically on identity‐related processes contributing to smoking relapse and did not assess other potential factors underlying relapse risk such as craving, stress, or environmental cues. However, alternative explanations for the observed associations cannot be ruled out. For example, self‐discontinuity may partly reflect broader psychological distress experienced during smoking cessation rather than a distinct psychological process. Likewise, fluctuations in smoker identity and relapse risk may be influenced by unmeasured factors that were beyond the scope of the present study. Future research should simultaneously examine identity‐related processes alongside other established predictors of smoking relapse to determine their unique contributions to relapse risk. Second, because a substantial proportion of participants had quit smoking several years prior to baseline, we restricted our analyses to individuals who had quit within the preceding 5 years. This decision was guided by empirical evidence (e.g. García‐Rodríguez et al., 2013) and by the need to retain a sufficiently large sample for longitudinal analyses. Nevertheless, a 5‐year timeframe may still be too broad to fully capture the identity‐related processes examined here. These are likely to be most pronounced during the first weeks and months following cessation, which also represents the period of greatest relapse risk. Consequently, our findings may not generalize to the earliest stages of smoking cessation. Future research should therefore investigate whether the present findings are replicated among more recent quitters. Third, participants were recruited through an online research platform, which may limit the generalizability of the findings. Replication in a more diverse community would therefore strengthen confidence in the robustness and validity of the present findings. Fourth, the multiple imputation procedure did not explicitly account for the nesting of repeated observations within participants. Although temporal dependencies were preserved when deriving lagged variables, the imputation itself was not formally multilevel.

### Conclusion

The present research highlighted the role of self‐discontinuity in understanding smoking relapse. Our findings indicated that within‐person increases in self‐discontinuity were associated with a higher likelihood of relapse through concurrent increases in smoker identity. In contrast, self‐discontinuity did not predict relapse through lagged changes in identity, and sensitivity analyses yielded no evidence for reverse indirect pathways whereby identity exerted indirect effects on relapse via self‐discontinuity. However, increases over time in both smoker and ex‐smoker identities preceded subsequent increases in self‐discontinuity, suggesting that identity fluctuations may precede disruptions in self‐continuity rather than the reverse. Taken together, these findings suggested that self‐discontinuity and smoker identity constitute closely related psychological factors associated with smoking relapse, although the temporal ordering of these factors warrants further investigation.

## CONFLICT OF INTEREST STATEMENT

The authors declare no conflicts of interest.

## ETHICS STATEMENT

The study received ethical approval from the institutional review board of our institution (CUREG‐2021‐10‐114). Informed consent was obtained from all participants prior to their participation in the study.

## Supporting information


**Table S1.** Sample demographic characteristics (baseline).
**Table S2.** Distribution of responses to the smoking status items across waves.
**Table S3.** Number and percentage of missing observations by variable and wave.
**Table S4.** Results of the partially prospective mediation model controlling for age, gender, and socioeconomic status (Model 1).
**Table S5.** Results of the fully prospective mediation model controlling for age, gender, and socioeconomic status (Model 2).
**Table S6.** Sensitivity analysis of the partially prospective model (Model 1) using current smoking status as an alternative outcome.
**Table S7.** Results of the partially prospective mediation model with non‐imputed data (Model 1).
**Table S8.** Longitudinal measurement invariance analyses.
**Figure S1.** Participant flow diagram across the four study waves.

## Data Availability

The dataset, analysis scripts, and the accompanying codebook are available at https://osf.io/3gwu4.

## ENDNOTES

1 Supplementary longitudinal measurement invariance analyses (configural, metric, and scalar) supported the longitudinal comparability of the self‐discontinuity, smoker identity, and ex‐smoker identity measures across waves (see Table S8 for details). However, it is important to note that these analyses were conducted using latent variables with individual items as observed indicators. In contrast, the primary analyses relied on observed composite scores in multilevel models.

2 A pilot study conducted with 88 ex‐smokers who had quit within the last 5 years was carried out prior to the main study to examine the psychometric properties of the self‐discontinuity scale. Participants completed several measures, including the self‐discontinuity scale and the smoker and ex‐smoker identity measures used in the present research. The self‐discontinuity scale demonstrated good internal consistency (M = 2.72, *SD* = 1.43, *α* = .90). A confirmatory factor analysis (CFA) conducted on the five self‐discontinuity items showed good fit to the data, *χ* ^2^(5) = 6.75, *p* = .240, root mean square error of approximation (RMSEA) = .063, standardized root mean square residual (SRMR) = .023, comparative fit index (CFI) = .995, with satisfactory standardized factor loadings ranging from .614 to .950 (all *p*s < .001). A second CFA including the self‐discontinuity, smoker identity, and ex‐smoker identity items indicated that a three‐factor model provided a better fit to the data, *χ* ^2^(38) = 72.87, *p* < .001, RMSEA = .103, SRMR = .074, CFI = .925, than a one‐factor model, *χ* ^2^(41) = 104.27, *p* < .001, RMSEA = .133, SRMR = .092, CFI = .864, providing support for the distinctiveness of self‐discontinuity from smoker and ex‐smoker identity.

3 We acknowledge that the present relapse operationalization encompasses different smoking trajectories (e.g. continued smoking and incident relapse). As a sensitivity analysis, we alternatively operationalized the outcome as current smoking status at the subsequent wave (1 = currently smoking, 0 = not currently smoking) and re‐estimated Model 1. Under this specification, the indirect effects were no longer statistically significant. Model 2 could not be reliably re‐estimated because the alternative outcome showed insufficient within‐person variation for the fully prospective multilevel specification. Detailed results are reported in Table S6.

## References

[aphw70231-bib-0001] Betty Ford Institute Consensus Panel . (2007). What is recovery? A working definition from the Betty Ford Institute. Journal of Substance Abuse Treatment, 33(3), 221–228. 10.1016/j.jsat.2007.06.001 17889294

[aphw70231-bib-0002] Blondé, J. , & Falomir‐Pichastor, J. M. (2020). Accounting for the negative consequences of tobacco dependence on smoking cravings, self‐efficacy, and motivation to quit: Consideration of identity concerns. The Spanish Journal of Psychology, 23, e34. 10.1017/SJP.2020.34 32895063

[aphw70231-bib-0003] Blondé, J. , & Falomir‐Pichastor, J. M. (2021). Tobacco dependence and motivation to quit smoking: An identity‐based framework. International Journal of Social Psychology, 36(2), 215–240. 10.1080/02134748.2021.1882224

[aphw70231-bib-0004] Blondé, J. , & Falomir‐Pichastor, J. M. (2026). Self‐discontinuity as a barrier to identity and behaviour change: Evidence from smoking cessation. European Journal of Social Psychology, 56(2), 316–330. 10.1002/ejsp.70044

[aphw70231-bib-0005] Breakwell, G. M. (1986). Coping with threatened identities. Methuen.

[aphw70231-bib-0006] Brown, T. A. (2015). Confirmatory factor analysis for applied research (2nd ed.). Guilford.

[aphw70231-bib-0007] Callaghan, L. , Yong, H. H. , Borland, R. , Cummings, K. M. , Hitchman, S. C. , & Fong, G. T. (2021). What kind of smoking identity following quitting would elevate smokers relapse risk? Addictive Behaviors, 112, 106654. 10.1016/j.addbeh.2020.106654 PMC7572756 32977267

[aphw70231-bib-0008] Chaiton, M. , Diemert, L. , Cohen, J. E. , Bondy, S. J. , Selby, P. , Philipneri, A. , & Schwartz, R. (2016). Estimating the number of quit attempts it takes to quit smoking successfully in a longitudinal cohort of smokers. BMJ Open, 6(6), e011045. 10.1136/bmjopen-2016-011045 PMC4908897 27288378

[aphw70231-bib-0009] Chandler, M. J. , & Proulx, T. (2008). Personal persistence and persistent people: Continuities in the lives of individuals and whole cultural communities. In F. Sani (Ed.), Self‐continuity: Individual and collective perspectives (pp. 213–226). Taylor & Francis.

[aphw70231-bib-0010] Curran, P. J. , & Bauer, D. J. (2011). The disaggregation of within‐person and between‐person effects in longitudinal models of change. Annual Review of Psychology, 62, 583–619. 10.1146/annurev.psych.093008.100356 PMC3059070 19575624

[aphw70231-bib-0011] Dijkstra, A. , & Borland, R. (2003). Residual outcome expectations and relapse in ex‐smokers. Health Psychology, 22(4), 340–346. 10.1037/0278-6133.22.4.340 12940389

[aphw70231-bib-0012] Elfeddali, I. , Bolman, C. , Candel, M. J. J. M. , Wiers, R. W. , & De Vries, H. (2012). The role of self‐efficacy, recovery self‐efficacy, and preparatory planning in predicting short‐term smoking relapse. British Journal of Health Psychology, 17(1), 185–201. 10.1111/j.2044-8287.2011.02032.x 22107073

[aphw70231-bib-0013] Falomir, J. M. , & Invernizzi, F. (1999). The role of social influence and smoker identity in resistance to smoking cessation. Swiss Journal of Psychology, 58(2), 73–84. 10.1024/1421-0185.58.2.73

[aphw70231-bib-0014] Falomir‐Pichastor, J. M. , Blondé, J. , Desrichard, O. , Felder, M. , Riedo, G. , & Folly, L. (2020). Tobacco dependence and smoking cessation: The mediating role of smoker and ex‐smoker self‐concepts. Addictive Behaviors, 102, 106200. 10.1016/j.addbeh.2019.106200 31801103

[aphw70231-bib-0015] Fiedler, K. , Harris, C. , & Schott, M. (2018). Unwarranted inferences from statistical mediation tests: An analysis of articles published in 2015. Journal of Experimental Social Psychology, 75, 95–102. 10.1016/j.jesp.2017.11.008

[aphw70231-bib-0016] García‐Rodríguez, O. , Secades‐Villa, R. , Flórez‐Salamanca, L. , Okuda, M. , Liu, S. M. , & Blanco, C. (2013). Probability and predictors of relapse to smoking: Results of the National Epidemiologic Survey on Alcohol and Related Conditions (NESARC). Drug and Alcohol Dependence, 132(3), 479–485. 10.1016/j.drugalcdep.2013.03.008 PMC3723776 23570817

[aphw70231-bib-0017] Hamaker, E. L. , Kuiper, R. M. , & Grasman, R. P. (2015). A critique of the cross‐lagged panel model. Psychological Methods, 20(1), 102–116. 10.1037/a0038889 25822208

[aphw70231-bib-0018] Herd, N. , Borland, R. , & Hyland, A. (2009). Predictors of smoking relapse by duration of abstinence: Findings from the International Tobacco Control (ITC) Four Country Survey. Addiction, 104(12), 2088–2099. 10.1111/j.1360-0443.2009.02732.x PMC4517970 19922574

[aphw70231-bib-0019] Hertel, A. W. , & Mermelstein, R. J. (2016). Smoker identity development among adolescents who smoke. Psychology of Addictive Behaviors, 30(4), 475–483. 10.1037/adb0000171 PMC4914471 27136374

[aphw70231-bib-0020] Hughes, J. R. , Solomon, L. J. , Naud, S. , Fingar, J. R. , Helzer, J. E. , & Callas, P. W. (2014). Natural history of attempts to stop smoking. Nicotine & Tobacco Research, 16(9), 1190–1198. 10.1093/ntr/ntu052 PMC4184396 24719491

[aphw70231-bib-0021] Iyer, A. , & Jetten, J. (2011). What's left behind: Identity continuity moderates the effect of nostalgia on well‐being and life choices. Journal of Personality and Social Psychology, 101(1), 94–108. 10.1037/a0022496 21355658

[aphw70231-bib-0022] Johnson, J. L. , Lovato, C. Y. , Maggi, S. , Ratner, P. A. , Shoveller, J. , Baillie, L. , & Kalaw, C. (2003). Smoking and adolescence: Narratives of identity. Research in Nursing & Health, 26(5), 387–397. 10.1002/nur.10102 14579259

[aphw70231-bib-0023] Kim, H. S. , Wohl, M. J. , Salmon, M. , & Santesso, D. (2017). When do gamblers help themselves? Self‐discontinuity increases self‐directed change over time. Addictive Behaviors, 64, 148–153. 10.1016/j.addbeh.2016.08.037 27614053

[aphw70231-bib-0024] Kim, H. S. , & Wohl, M. J. A. (2015). The bright side of self‐discontinuity: Feeling disconnected with the past self increases readiness to change addictive behaviors (via nostalgia). Social Psychological and Personality Science, 6(2), 229–237. 10.1177/1948550614549482

[aphw70231-bib-0025] Meijer, E. , Gebhardt, W. A. , Dijkstra, A. , Willemsen, M. C. , & Van Laar, C. (2015). Quitting smoking: The importance of non‐smoker identity in predicting smoking behaviour and responses to a smoking ban. Psychology & Health, 30(12), 1387–1409. 10.1080/08870446.2015.1049603 25959600

[aphw70231-bib-0026] Meijer, E. , Van den Putte, B. , Gebhardt, W. A. , Van Laar, C. , Bakk, Z. , Dijkstra, A. , Fong, G. T. , West, R. , & Willemsen, M. C. (2018). A longitudinal study into the reciprocal effects of identities and smoking behaviour: Findings from the ITC Netherlands Survey. Social Science & Medicine (1982), 200, 249–257. 10.1016/j.socscimed.2017.12.006 29321102

[aphw70231-bib-0027] Meijer, E. , van Laar, C. , Gebhardt, W. A. , Fokkema, M. , van den Putte, B. , Dijkstra, A. , Fong, G. T. , & Willemsen, M. C. (2017). Identity change among smokers and ex‐smokers: Findings from the ITC Netherlands Survey. Psychology of Addictive Behaviors, 31(4), 465–478. 10.1037/adb0000281 28493751

[aphw70231-bib-0028] Meijer, E. , Vangeli, E. , Gebhardt, W. A. , & van Laar, C. (2020). Identity processes in smokers who want to quit smoking: A longitudinal interpretative phenomenological analysis. Health, 24(5), 493–517. 10.1177/1363459318817923 30541353

[aphw70231-bib-0029] Notley, C. , & Colllins, R. (2018). Redefining smoking relapse as recovered social identity—Secondary qualitative analysis of relapse narratives. Journal of Substance Use, 23(6), 660–666. 10.1080/14659891.2018.1489009

[aphw70231-bib-0030] Partos, T. R. , Borland, R. , Yong, H. , Hyland, A. , & Cummings, K. M. (2013). The quitting rollercoaster: How recent quitting history affects future cessation outcomes (data from the International Tobacco Control 4‐Country Cohort Study). Nicotine & Tobacco Research, 15(9), 1578–1587. 10.1093/ntr/ntt025 PMC3741062 23493370

[aphw70231-bib-0031] Poole, R. , Carver, H. , Anagnostou, D. , Edwards, A. , Moore, G. , Smith, P. , Wood, F. , & Brain, K. (2022). Tobacco use, smoking identities and pathways into and out of smoking among young adults: A meta‐ethnography. Substance Abuse Treatment, Prevention, and Policy, 17(1), 24. 10.1186/s13011-022-00451-9 PMC8960094 35346260

[aphw70231-bib-0032] Rutchick, A. M. , Slepian, M. L. , Reyes, M. O. , Pleskus, L. N. , & Hershfield, H. E. (2018). Future self‐continuity is associated with improved health and increases exercise behavior. Journal of Experimental Psychology: Applied, 24(1), 72–80. 10.1037/xap0000153 29595304

[aphw70231-bib-0033] Sani, F. (2008). Self‐continuity: Individual and collective perspectives. Psychology Press.

[aphw70231-bib-0034] Shadel, W. G. , & Mermelstein, R. (1996). Individual differences in self‐concept among smokers attempting to quit: Validation and predictive utility of measures of the smoker self‐concept and abstainer self‐concept. Annals of Behavioral Medicine, 18(3), 151–156. 10.1007/BF02883391 24203766

[aphw70231-bib-0035] Shiffman, S. , Balabanis, M. H. , Paty, J. A. , Engberg, J. , Gwaltney, C. J. , Liu, K. S. , Gnys, M. , Hickcox, M. , & Paton, S. M. (2000). Dynamic effects of self‐efficacy on smoking lapse and relapse. Health Psychology, 19(4), 315–323. 10.1037//0278-6133.19.4.315 10907649

[aphw70231-bib-0036] Tombor, I. , Shahab, L. , Herbec, A. , Neale, J. , Michie, S. , & West, R. (2015). Smoker identity and its potential role in young adults' smoking behavior: A meta‐ethnography. Health Psychology, 34(10), 992–1003. 10.1037/hea0000191 PMC4577249 25622078

[aphw70231-bib-0037] van den Putte, B. , Yzer, M. , Willemsen, M. C. , & de Bruijn, G. J. (2009). The effects of smoking self‐identity and quitting self‐identity on attempts to quit smoking. Health Psychology, 28(5), 535–544. 10.1037/a0015199 19751079

[aphw70231-bib-0038] Vangeli, E. , Stapleton, J. , & West, R. (2010). Residual attraction to smoking and smoker identity following smoking cessation. Nicotine & Tobacco Research, 12(8), 865–869. 10.1093/ntr/ntq104 20622025

[aphw70231-bib-0039] Vangeli, E. , & West, R. (2012). Transition towards a ‘non‐smoker’ identity following smoking cessation: An interpretative phenomenological analysis. British Journal of Health Psychology, 17(1), 171–184. 10.1111/j.2044-8287.2011.02031.x 22107052

[aphw70231-bib-0040] Vignoles, V. L. , Regalia, C. , Manzi, C. , Golledge, J. , & Scabini, E. (2006). Beyond self‐esteem: Influence of multiple motives on identity construction. Journal of Personality and Social Psychology, 90(2), 308–333. 10.1037/0022-3514.90.2.308 16536653

[aphw70231-bib-0041] West, R. (2006). Theory of addiction. Blackwell, Addiction Press.

[aphw70231-bib-0042] Wohl, M. J. A. , Kim, H. S. , Salmon, M. , Santesso, D. , Wildschut, T. , & Sedikides, C. (2018). Discontinuity‐induced nostalgia improves the odds of a self‐reported quit attempt among people living with addiction. Journal of Experimental Social Psychology, 75, 83–94. 10.1016/j.jesp.2017.11.011

[aphw70231-bib-0043] Zhao, X. , Dichtl, F. F. , & Foran, H. M. (2022). Predicting smoking behavior: Intention and future self‐continuity among Austrians. Psychology, Health & Medicine, 27(5), 1042–1051. 10.1080/13548506.2020.1842898 33147066

[aphw70231-bib-0044] Zhou, X. , Nonnemaker, J. , Sherrill, B. , Gilsenan, A. W. , Coste, F. , & West, R. (2009). Attempts to quit smoking and relapse: Factors associated with success or failure from the ATTEMPT cohort study. Addictive Behaviors, 34(4), 365–373. 10.1016/j.addbeh.2008.11.013 19097706

