# Supplementary material for: Self‐discontinuity, smoker and ex‐smoker identity, and smoking relapse: A multilevel longitudinal study
Source: Appl Psychol Health Well Being. 2026 Sep 25;18(5):e70231. doi: 10.1111/aphw.70231 (PMC13614110; doi:10.1111/aphw.70231)
Supplement: Supplementary file 1 [file APHW-18-0-s001.docx]

**Supplementary materials**

**Self-Discontinuity, Smoker and Ex-Smoker Identity, and Smoking Relapse:**

**A Multilevel Longitudinal Study**

**Table S1.**

**Sample demographic characteristics (baseline)**

|  | Mean (*SD*) | *n* (%) | *N* |
| --- | --- | --- | --- |
| Gender |  |  |  |
| Female |  | 49.2 | 218 |
| Male |  | 47.6 | 211 |
| Other |  | 1.6 | 7 |
| Age | 39.91 (11.87) |  |  |
| Employment |  |  |  |
| In paid work |  | 77.0 | 341 |
| In education |  | 2.0 | 9 |
| Unemployed |  | 4.5 | 20 |
| Permanently sick/disabled |  | 4.5 | 20 |
| Retired |  | 4.3 | 19 |
| Other |  | 9.9 | 27 |
| Education |  |  |  |
| Post-graduate degree |  | 17.4 | 77 |
| College graduation |  | 48.5 | 215 |
| High school graduation |  | 30.0 | 133 |
| Less than high school graduation |  | 1.6 | 7 |
| No qualifications |  | 0.9 | 4 |
| Income^1^ |  |  |  |
| More than 70,000 |  | 10.4 | 46 |
| 50,000 to 70,000 |  | 16.2 | 72 |
| 30,000 to 50,000 |  | 27.8 | 123 |
| 10,000 to 30,000 |  | 36.6 | 162 |
| Less than 10,000 |  | 7.5 | 33 |
| Country of residence |  |  |  |
| UK |  | 87.4 | 387 |
| US |  | 11.1 | 49 |
| Past smoking consumption (cig/day) | 13.56 (8.49) |  |  |
| Duration of current quit attempt (months) | 26.32 (18.22) |  |  |
| Smoking duration (years) | 15.61 (10.73) |  |  |
| Past quit attempts |  |  |  |
| Yes |  | 80.4 | 356 |
| No |  | 19.6 | 87 |
| Number of quit attempts | 6.39 (11.02) |  |  |

*Notes*. Differences between the reported sample sizes and the total sample size (*N* = 443) are due to missing data;

^1^Because participants were recruited in both the UK and the US, income was reported in either British pounds (GBP) or US dollars (USD). Each response category was presented using both currencies (e.g., “£10,001–£15,000 / $10,001–$15,000”), and participants were instructed to select the category corresponding to their income in their local currency. These categories were intended to provide a broad indicator of income level and should not be interpreted as representing equivalent income or purchasing power across the two countries. No currency conversion was performed.

**Table S2.**

Distribution of responses to the smoking status items across waves

| **Wave** | **Response options** | ***n*** | **%** | **Coding** |
| --- | --- | --- | --- | --- |
| **Wave 2** | “*I did not smoke at the time of the last questionnaire, I have smoked cigarettes again since then, but I am not currently smoking anymore*” | 81 | 22.9 | Relapse |
|  | “*I did not smoke at the time of the last questionnaire, I have smoked cigarettes again since then, and I am still currently smoking*” | 13 | 3.7 | Relapse |
|  | “*I did not smoke at the time of the last questionnaire, and I have not smoked a single cigarette since then*” | 260 | 73.4 | No relapse |
| **Wave 3** | “*I used to smoke at the time of the last questionnaire, and I have not attempted to quit since then*” | 7 | 2.0 | Relapse |
|  | “*I used to smoke at the time of the last questionnaire, I have attempted to quit since then, but I am currently smoking again*” | 14 | 4.1 | Relapse |
|  | “*I used to smoke at the time of the last questionnaire, I have attempted to quit since then, and I am still currently not smoking*” | 19 | 5.5 | Relapse |
|  | “*I did not smoke at the time of the last questionnaire, I have smoked cigarettes again since then, but I am not currently smoking anymore*” | 57 | 16.6 | Relapse |
|  | “*I did not smoke at the time of the last questionnaire, I have smoked cigarettes again since then, and I am still currently smoking*” | 7 | 1.6 | Relapse |
|  | “*I did not smoke at the time of the last questionnaire, and I have not smoked a single cigarette since then*” | 240 | 69.8 | No relapse |
| **Wave 4** | “*I used to smoke at the time of the last questionnaire, and I have not attempted to quit since then*” | 10 | 3.2 | Relapse |
|  | “*I used to smoke at the time of the last questionnaire, I have attempted to quit since then, but I am currently smoking again*” | 18 | 5.7 | Relapse |
|  | “*I used to smoke at the time of the last questionnaire, I have attempted to quit since then, and I am still currently not smoking*” | 17 | 5.4 | Relapse |
|  | “*I did not smoke at the time of the last questionnaire, I have smoked cigarettes again since then, but I am not currently smoking anymore*” | 54 | 17.1 | Relapse |
|  | “*I did not smoke at the time of the last questionnaire, I have smoked cigarettes again since then, and I am still currently smoking*” | 4 | 1.3 | Relapse |
|  | “*I did not smoke at the time of the last questionnaire, and I have not smoked a single cigarette since then*” | 213 | 67.4 | No relapse |

**Table S3.**

Number and percentage of missing observations by variable and wave

| **Variable** | **Missing**  **= count** | **Missing**  **= percent** |
| --- | --- | --- |
| Discontinuity T1 | 0 | 0 |
| Discontinuity T2 | 106 | 23.9 |
| Discontinuity T3 | 127 | 28.7 |
| Discontinuity T4 | 160 | 36.1 |
| Smoker identity T1 | 0 | 0 |
| Smoker identity T2 | 93 | 21.0 |
| Smoker identity T3 | 101 | 22.8 |
| Smoker identity T4 | 128 | 28.9 |
| Ex-smoker identity T1 | 0 | 0 |
| Ex-smoker identity T2 | 93 | 21.0 |
| Ex-smoker identity T3 | 101 | 22.8 |
| Ex-smoker identity T4 | 128 | 28.9 |
| Relapse T2 | 89 | 20.1 |
| Relapse T3 | 99 | 22.3 |
| Relapse T4 | 127 | 28.7 |

*Note.* Percentages are based on the baseline sample (*N* = 443).

**Table S4.**

Results of the partially prospective mediation model controlling for age, gender, and socioeconomic status (Model 1)

|  | *β* (*SE*) | *p* | 95% *CI* |
| --- | --- | --- | --- |
| **Within-person** |  |  |  |
| ***Auto-regressive path*** |  |  |  |
| Relapse | .253 (.10) | .010 | [.061, .446] |
| ***Direct associations*** |  |  |  |
| Self-discontinuity (*t*) → Smoker identity (*t*) | .277 (.02) | <.001 | [.241, .307] |
| Self-discontinuity (*t*) → Ex-smoker identity (*t*) | .094 (.02) | <.001 | [.052, .129] |
| Self-discontinuity (*t*) → Relapse (*t+1*) | -.049 (.09) | .579 | [-.223, .125] |
| Smoker identity (*t*) → Relapse (*t+1*) | .189 (.08) | .014 | [.038, .340] |
| Ex-smoker identity (*t*) → Relapse (*t+1*) | .116 (.08) | .142 | [-.039, .270] |
| ***Indirect effects*** |  |  |  |
| Self-discontinuity (*t*) → Smoker identity (*t*) → Relapse (*t+1*) | .040 (.02) | .017 | [.007, .074] |
| Self-discontinuity (*t*) → Ex-smoker identity (*t*) → Relapse (*t+1*) | .008 (.01) | .168 | [-.004, .020] |
| Total indirect effect | .050 (.02) | .007 | [.013, .084] |
| **Between-person** |  |  |  |
| Quit attempt duration | -.375 (.06) | <.001 | [-.500, -.250] |
| Smoking duration | .045 (.09) | .646 | [-.146, .235] |
| Past smoking consumption | -.086 (.07) | .195 | [-.216, .044] |
| Gender | .091 (.06) | .150 | [-.033, .216] |
| Age | -.344 (.09) | <.001 | [-.528, -.161] |
| Income | -.024 (.06) | .696 | [-.146, .097] |
| Education | .016 (.07) | .811 | [-.113, .144] |

*Notes.* Coefficients represent standardized within-person estimates from a two-level model. The model is partially prospective, with contemporaneous predictors and mediators measured at time *t* and smoking relapse measured at the subsequent wave (*t*+1).

**Table S5.**

Results of the fully prospective mediation model controlling for age, gender, and socioeconomic status (Model 2)

|  | *β* (*SE*) | *p* | 95% *CI* |
| --- | --- | --- | --- |
| **Within-person** |  |  |  |
| ***Auto-regressive paths*** |  |  |  |
| Self-discontinuity | .534 (.02) | <.001 | [.491, .577] |
| Smoker identity | .702 (.02) | <.001 | [.670, .734] |
| Ex-smoker identity | .622 (.02) | <.001 | [.588, .656] |
| Relapse | .412 (.05) | <.001 | [.316, .509] |
| ***Direct associations*** |  |  |  |
| Self-discontinuity (*t–1*) → Smoker identity (*t*) | .001 (.03) | .996 | [-.051, .051] |
| Self-discontinuity (*t–1*) → Ex-smoker identity (*t*) | -.023 (.03) | .401 | [-.078, .022] |
| Self-discontinuity (*t–1*) → Relapse (*t+1*) | -.027 (.18) | .879 | [-.380, .269] |
| Smoker identity (*t*) → Relapse (*t+1*) | .309 (.14) | .025 | [.040, .534] |
| Ex-smoker identity (*t*) → Relapse (*t+1*) | .082 (.12) | .508 | [-.161, .287] |
| ***Indirect effects*** |  |  |  |
| Self-discontinuity (*t–1*) → Smoker identity (*t*) → Relapse (*t+1*) | .001 (.01) | 1.000 | [-.012, .010] |
| Self-discontinuity (*t–1*) → Ex-smoker identity (*t*) → Relapse (*t+1*) | -.001 (.01) | .613 | [-.007, .003] |
| Total indirect effect | -.001 (.01) | .826 | [-.015, .012] |
| **Between-person** |  |  |  |
| Quit attempt duration | -.369 (.06) | <.001 | [-.490, -.248] |
| Smoking duration | -.034 (.10) | .724 | [-.157, .225] |
| Past smoking consumption | -.102 (.06) | .108 | [-.227, .023] |
| Gender | .079 (.06) | .195 | [-.041, .199] |
| Age | -.278 (.10) | .004 | [-.466, -.089] |
| Income | .052 (.06) | .393 | [-.113, .119] |
| Education | -.003 (.06) | .960 | [-.068, .172] |

*Notes.* Coefficients represent standardized within-person estimates from a two-level model. The model is fully prospective, with lagged predictors measured at the previous wave (*t*–1), mediators measured at the current wave (*t*), and smoking relapse measured at the subsequent wave (*t*+1).

**Table S6.**

Sensitivity analysis of the partially prospective model (Model 1) using current smoking status as an alternative outcome

|  | *β* (*SE*) | *p* | 95% *CI* |
| --- | --- | --- | --- |
| **Within-person** |  |  |  |
| ***Auto-regressive path*** |  |  |  |
| Relapse | .591 (.04) | <.001 | [.510, .673] |
| ***Direct associations*** |  |  |  |
| Self-discontinuity (*t*) → Smoker identity (*t*) | .273 (.02) | <.001 | [.235, .311] |
| Self-discontinuity (*t*) → Ex-smoker identity (*t*) | .106 (.02) | <.001 | [.062, .150] |
| Self-discontinuity (*t*) → Relapse (*t+1*) | .024 (.07) | .734 | [-.113, .161] |
| Smoker identity (*t*) → Relapse (*t+1*) | .139 (.08) | .077 | [-.015, .294] |
| Ex-smoker identity (*t*) → Relapse (*t+1*) | .044 (.06) | .475 | [-.077, .166] |
| ***Indirect effects*** |  |  |  |
| Self-discontinuity (*t*) → Smoker identity (*t*) → Relapse (*t+1*) | .036 (.02) | .090 | [-.006, .071] |
| Self-discontinuity (*t*) → Ex-smoker identity (*t*) → Relapse (*t+1*) | .004 (.01) | .482 | [-.008, .017] |
| Total indirect effect | .041 (.02) | .068 | [.003, .077] |
| **Between-person** |  |  |  |
| Quit attempt duration | -010. (.01) | .084 | [-.020, .001] |
| Smoking duration | -.015 (.01) | .175 | [-.037, .007] |
| Past smoking consumption | -.005 (.01) | .705 | [-.030, .020] |

*Notes.* Coefficients represent standardized within-person estimates from a two-level model. The model is partially prospective, with contemporaneous predictors and mediators measured at time *t* and smoking relapse measured at the subsequent wave (*t*+1).

**Table S7.**

Results of the partially prospective mediation model with non-imputed data (Model 1)

|  | *β* (*SE*) | *p* | 95% *CI* |
| --- | --- | --- | --- |
| **Within-person** |  |  |  |
| ***Auto-regressive path*** |  |  |  |
| Relapse | .601 (.04) | <.001 | [.530, .672] |
| ***Direct associations*** |  |  |  |
| Self-discontinuity (*t*) → Smoker identity (*t*) | .273 (.02) | <.001 | [.235, .311] |
| Self-discontinuity (*t*) → Ex-smoker identity (*t*) | .108 (.02) | <.001 | [.065, .151] |
| Self-discontinuity (*t*) → Relapse (*t+1*) | .016 (.06) | .801 | [-.105, .136] |
| Smoker identity (*t*) → Relapse (*t+1*) | .097 (.06) | .095 | [-.017, .211] |
| Ex-smoker identity (*t*) → Relapse (*t+1*) | .091 (.05) | .080 | [-.011, .193] |
| ***Indirect effects*** |  |  |  |
| Self-discontinuity (*t*) → Smoker identity (*t*) → Relapse (*t+1*) | .025 (.02) | .103 | [-.005, .056] |
| Self-discontinuity (*t*) → Ex-smoker identity (*t*) → Relapse (*t+1*) | .009 (.01) | .105 | [-.002, .021] |
| Total indirect effect | .035 (.02) | .031 | [.003, .066] |
| **Between-person** |  |  |  |
| Quit attempt duration | -.014 (.01) | .004 | [-.024, -.005] |
| Smoking duration | -.019 (.01) | .038 | [-.037, -.001] |
| Past smoking consumption | .004 (.01) | .637 | [-.014, .023] |

*Notes.* Coefficients represent standardized within-person estimates from a two-level model. The model is partially prospective, with contemporaneous predictors and mediators measured at time *t* and smoking relapse measured at the subsequent wave (*t*+1).

**Table S8.**

Longitudinal measurement invariance analyses

|  | *χ*2 | *df* | RMSEA | RMSEA 90% CI | sRMR | CFI |
| --- | --- | --- | --- | --- | --- | --- |
| **Configural** | 1624.51 | 770 | .050 | [.047 – .053] | .094 | .92 |
| **Metric** | 1651.91 | 794 | .049 | [.046 – .053] | .094 | .92 |
| **Scalar** | 1689.12 | 818 | .049 | [.046 – .052] | .095 | .92 |

**Figure S1.**

Participant flow diagram across the four study waves
